# Fluvastatin Reduces Glucose Tolerance in Healthy Young Individuals Independently of Cold Induced BAT Activity

**DOI:** 10.3389/fendo.2021.765807

**Published:** 2021-11-10

**Authors:** Martina Felder, Claudia Irene Maushart, Gani Gashi, Jaël Rut Senn, Anton S. Becker, Julian Müller, Miroslav Balaz, Christian Wolfrum, Irene A. Burger, Matthias Johannes Betz

**Affiliations:** ^1^Department of Endocrinology, Diabetes and Metabolism, University Hospital Basel and University of Basel, Basel, Switzerland; ^2^Institute of Diagnostic and Interventional Radiology, University Hospital Zurich/University of Zurich, Zurich, Switzerland; ^3^Institute of Food, Nutrition and Health, ETH Zurich, Zurich, Switzerland

**Keywords:** diabetes, brown adipose tissue, energy expenditure (EE), glucose tolerance, fluvastatin, statin, cold-induced thermogenesis

## Abstract

**Background:**

Statins are commonly prescribed for primary and secondary prevention of atherosclerotic disease. They reduce cholesterol biosynthesis by inhibiting hydroxymethylglutaryl-coenzyme A-reductase (HMG-CoA-reductase) and therefore mevalonate synthesis. Several studies reported a small, but significant increase in the diagnosis of diabetes mellitus with statin treatment. The molecular mechanisms behind this adverse effect are not yet fully understood. Brown adipose tissue (BAT), which plays a role in thermogenesis, has been associated with a reduced risk of insulin resistance. Statins inhibit adipose tissue browning and have been negatively linked to the presence of BAT in humans. We therefore speculated that inhibition of BAT by statins contributes to increased insulin resistance in humans.

**Methods:**

A prospective study was conducted in 17 young, healthy men. After screening whether significant cold-induced thermogenesis (CIT) was present, participants underwent glucose tolerance testing (oGTT) and assessment of BAT activity by FDG-PET/MRI after cold-exposure and treatment with a β3-agonist. Fluvastatin 2x40mg per day was then administered for two weeks and oGTT and FDG-PET/MRI were repeated.

**Results:**

Two weeks of fluvastatin treatment led to a significant increase in glucose area under the curve (AUC) during oGTT (p=0.02), reduction in total cholesterol and LDL cholesterol (both p<0.0001). Insulin AUC (p=0.26), resting energy expenditure (REE) (p=0.44) and diet induced thermogenesis (DIT) (p=0.27) did not change significantly. The Matsuda index, as an indicator of insulin sensitivity, was lower after fluvastatin intake, but the difference was not statistically significant (p=0.09). As parameters of BAT activity, mean standard uptake value (SUV_mean_) (p=0.12), volume (p=0.49) and total glycolysis (p=0.74) did not change significantly during the intervention. Matsuda index, was inversely related to SUV_mean_ and the respiratory exchange ratio (RER) (both R^2 =^ 0.44, p=0.005) at baseline, but not after administration of fluvastatin (R^2 =^ 0.08, p=0.29, and R^2 =^ 0.14, p=0.16, respectively).

**Conclusions:**

Treatment with fluvastatin for two weeks reduced serum lipid levels but increased glucose AUC in young, healthy men, indicating reduced glucose tolerance. This was not associated with changes in cold-induced BAT activity.

## Introduction

Statins are widely used drugs for primary and secondary prevention of atherosclerotic disease (1, 2). The effect of statins is based on the inhibition of hydroxymethylglutaryl-coenzyme A (HMG-CoA) reductase, which leads to a decrease in cholesterol biosynthesis (3). As a consequence, hepatocytes increase the number of LDL-receptors, thereby clearing more LDL particles from the blood stream (4). Statins are highly effective, generally well tolerated (5) and have an excellent risk-benefit ratio (6). However, as compared to placebo, a small but significant proportion of patients treated with statins gets diagnosed with diabetes (7, 8). Studies investigating the effect of statins on human glucose metabolism described effects on both insulin sensitivity and insulin secretion (7, 9). The molecular mechanisms behind this adverse effect are still being investigated. We previously demonstrated that statin use was negatively associated with the presence of brown adipose tissue (BAT) in humans undergoing routine ^18^F-FDG-PET/CT scanning. Furthermore, we were able to show that statins inhibit geranylgeranylation of small GTP-binding proteins, thereby impeding adipose tissue browning (10).

BAT is athermogenic organ and unique in its ability to directly convert chemical energy from lipids or carbohydrates into heat by short-circuiting the respiratory chain. It plays an important role in maintaining core body temperature when newborn mammals or rodents are exposed to cold environments (11). In humans, activated BAT contributes significantly to whole body energy metabolism. It plays a major role in thermogenesis and is mainly found in infants and children (12). Active BAT requires glucose and free fatty acids (FFAs) as substrates for thermogenesis (11). Thus, thermogenic BAT may play an important role in systemic glucose and lipid homeostasis (13). Several studies have shown that BAT activity is maintained in adults but decreases as people age (14, 15). In contrast to white adipose tissue (WAT), BAT contains an abundance of mitochondria, more capillaries and is more densely innervated by the sympathetic nervous system (SNS) (16). When exposed to cold temperatures, the SNS activates brown adipocytes *via* its main transmitter norepinephrine, leading to rapid lipolysis. The resulting FFAs activate uncoupling protein 1 (UCP1) in the inner mitochondrial membrane (IMM). This reduces the proton gradient across the IMM resulting in the production of heat (17). The subsequent release of heat enables efficient thermogenesis and is called cold-induced thermogenesis (CIT), which can be measured by indirect calorimetry (18). In general, the presence of active BAT has been associated with higher energy expenditure (EE), lower proportion of body fat and reduced risk of insulin resistance (19, 20)

(10) We speculated that inhibition of BAT activity by statins contributes to enhanced insulin resistance, thereby increasing the incidence of type 2 diabetes in statin-treated patients. We investigated the effect of different statins on BAT differentiation *in vitro* and in mice. Fluvastatin (21) inhibited the differentiation of human multipotent adipose stem cells into brown adipocytes more potently than the modern statins atorvastatin and rosuvastatin (10). This might be due to the fact that fluvastatin is lipophilic and has a much lower liver selectivity, as compared to the hydrophilic statins (22). Therefore, we conducted a small prospective clinical trial assessing the effect of fluvastatin on human BAT and glucose tolerance in healthy volunteers. Our objective was to investigate whether fluvastatin leads to increased insulin resistance, due to an inhibitory effect on BAT in human adults and if changes in glycemic control are related to parameters of BAT activity. Additionally, we wanted to assess whether taking fluvastatin affects diet induced thermogenesis (DIT) or CIT.

## Methods

### Subjects and Screening

**Figure 1:**
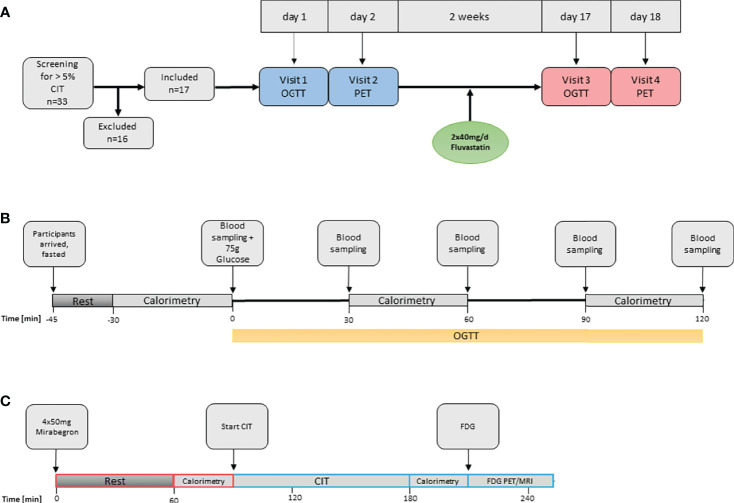
Overview of study procedures. **(A)** Overall study flow. CIT, cold induced thermogenesis; OGTT, oral glucose tolerance test. **(B)** Oral glucose tolerance test and assessment of diet-induced thermogenesis. **(C)** Assessment of BAT function. FDG, Fluorodeoxyglucose.

This study is part of the FluvaBAT trial (clinicaltrials.gov ID NCT03189511) which prospectively investigated the effect of fluvastatin on human BAT (10). The Ethics Committee for Northwestern and Central Switzerland (EKNZ, Number 2017-00455) approved the study and all participants provided written informed consent. Healthy, male volunteers were screened for presence of significant CIT and 17 participants were enrolled in the trial. Sixteen participants completed the trial. One participant was excluded from the analysis after showing no significant BAT activity in the PET. An overview of the trial flow is provided in **Figure 1A**.

### Study Design

The study was designed as an open-label before-and-after trial assessing the effect of 40 mg of fluvastatin twice daily on glucose tolerance, CIT and BAT activity. Participants attended a screening visit and two study visits, each before and after two weeks of treatment. At the screening visit CIT was assessed and only participants with a CIT > 5% were enrolled into the study. Within two weeks of the screening visit, an oral glucose tolerance test (oGTT) was performed and on the following day BAT activity was assessed by [^18^F]-FDG-PET/MRI after cold-stimulation. Thereafter, the participants took fluvastatin for 14 days and identical visits were performed at the end of the treatment period.

### Oral Glucose Tolerance Test

Participants arrived fasted (no caloric intake for at least six hours) in the morning of the study day. They were asked to refrain from strenuous exercise for at least 24 hours prior to the study visit. After an initial resting phase of at least 15 minutes an indirect calorimetry was performed with the participant lying on a hospital bed. After a baseline blood sampling oGTT was performed: All participants drank 75 g of glucose dissolved in 250 ml tap water. Blood was sampled at 30, 60, 90 and 120 minutes after ingestion of glucose. Note, that for the first seven patients’ blood was only sampled at 0, 60 and 120 minutes. EE was measured three times during visit 1 and visit 3: just before, as well as 30 to 60 and 90 to 120 minutes after the intake of 75 g glucose, by indirect calorimetry (**Figure 1B**).

### Laboratory Analysis

We determined plasma glucose, sodium, potassium, kidney and liver parameters, TSH, fT4 and vitamin D at the screening visit to rule out metabolic disorders and to check the kidney function in order to ensure the safety of the study participants. Total cholesterol, LDL and triglycerides were measured before and after two weeks of fluvastatin in order to monitor compliance with the study drug. Glucose and insulin values were determined during the oGTT at the time points specified above. All routine analysis were carried out at the central laboratory of the University Hospital Basel. Cholesterol, HDL, triglycerides and glucose levels were measured using enzymatic methods on a COBAS 8800 analyzer (Roche Diagnostics, Mannheim, Germany). LDL levels were calculated using the Friedewald formula. Insulin levels were measured using an immuno-assay (ECLIA, Roche Diagnostics).

### Measurement of Fibroblast Growth Factor 21

Fibroblast growth factor 21 (FGF21) levels were measured by enzyme-linked immunosorbent assay with a sensitivity of 8.69 pg/mL and an intra-assay coefficient of variation (CV) of 2.9 to 3.9% and an inter-assay CV of 5.2 to 10.9% (range given in product data sheet, Human FGF-21 Quantikine ELISA Kit, R&D Systems, Minneapolis, MN). In all 16 participants who completed the study, samples were taken at 0 min and 120 min after ingestion of 75 g glucose.

### Indirect Calorimetry

Indirect calorimetry was performed over a period of 30 minutes with the participants resting comfortably in a supine position on a hospital bed using a Quark RMR (Cosmed, Rome, Italy). All measurements took place in an air-conditioned examination room at a constant temperature of 24°C. The subjects were asked to fast for at least 6 h before the visit, and to abstain from exercise 24 h before, as these factors are known to affect the sensitivity of the measurement system. The sensitivity of an indirect calorimeter to detect significant changes in energy expenditure is approximately 1.5% (23). The calorimeter sensors were calibrated prior to each study visit and the system has previously been shown to perform well in comparison to “gold-standard” calorimeters (24).

### Cold Induced Thermogenesis

CIT was calculated as the difference between resting energy expenditure (REE) at warm ambient temperatures and EE after a mild cold stimulus of two hours duration, as described previously (25). To determine CIT at the screening visit, participants were resting comfortably on a hospital bed, wearing t-shirts and shorts and were covered with a fleece blanket. After baseline indirect calorimetry and measurement of temperature, the blanket was removed and a water-circulating cooling system (Hilotherm Clinic, Hilotherm GmbH, Germany) was placed around the waist. Every 2 min the water temperature was reduced by 1°C, starting from 25°C to a minimum of 10°C. Cooling time was 120 minutes and care was taken to ensure that subjects did not shiver. When shivering occurred, the water temperature was raised by 2°C and the participant was covered with a fleece blanket until the shivering stopped. During the last 30 min of cold exposure, a second measurement of EE was performed and temperature was taken again.

### Assessment of BAT Activity by FDG-PET/MRI

As the β3-adrenoreceptor agonist mirabegron had been recently demonstrated to activate human BAT (26), we decided to combine cold exposure and β3 adrenergic stimulation as to assess the maximum metabolic activity of BAT. Subjects arrived in a fasted state in the morning of the study day. After the intake of 4 x 50 mg Mirabegron, the participants were instructed to wait for 1.5 hours, followed by 2 hours of standardized cooling as described above. Before start of the dynamic [^18^F]-FDG-PET/MRI Scan, 75MBq [^18^F]-FDG was injected through an intravenous line. Total scan time was 40min with a partial body scan from head to the upper abdomen after 30 minutes (**Figure 1C**).

### Segmentation of BAT

For the quantification of glucose uptake into BAT, we used the 3D Slicer software, version 4.11.20 (National Institutes of Health, Bethesda, MD). In order to reliably segment supraclavicular adipose tissue (AT) for measurement of fat-fraction (FF) and glucose uptake, we used a step-wise segmentation approach (27). In brief, it consisted of a rough anatomic segmentation of the supraclavicular AT depot, which was further refined by thresholding based on the FF in the MRI volume and the [^18^F]-FDG uptake in the PET volume.

The suspected area containing the AT was outlined in the neck and shoulder region with the sphere brush tool of 3D slicer. In a next step, we used the FF volume and included all voxels with a FF of 400-1000‰ as further segmentation. The generated segments were intersected to create the volume of interest, MRI and PET volumes were co-registered (PET/MRI) and therefore the corresponding PET image could be overlaid.

### Statistical Analysis

Data analysis was performed using GraphPad Prism Version 9 (GraphPad Inc., La Jolla, CA). Pairwise comparisons were calculated, using paired t tests. Repeated measures were analyzed by two-way ANOVA. Correlations were first analyzed by simple linear regression. Mixed-effects models were built in R Version 4.1.0 (28) using the “nlme” library (29) to investigate co-variables influencing insulin sensitivity. As levels of FGF21 are usually not normally distributed, they were log transformed prior to statistical analysis.

## Results

### Baseline Characteristics

We saw 16 healthy male volunteers both before and after administration of fluvastatin 2x40 mg b.i.d. for 14 days. Baseline characteristics of the participants are summarized in **Table 1**.

**Table 1:** Characteristics of the study participants.

	Means ± SD
Number	17
Age, yr	23.7 ± 3.6
Weight, kg	76.9 ± 5.7
Height, cm	182.1 ± 6.1
BMI, kg/m^2^	23.2 ± 1.7
Systolic blood pressure, mmHg	124.9 ± 11.3
Diastolic blood pressure, mmHg	66.4 ± 7.6
Heart rate, bpm	66.4 ± 13.2
Fasting glucose, mM	4.7 ± 0.4
HbA1c, %	5.0 ± 0.2
Sodium, mM	139.8 ± 1.4
Potassium, mM	3.9 ± 0.3
Creatinine, µM	84.9 ± 11.5
Urea, mM	4.7 ± 1.1
ASAT, U/l	26.5 ± 5.5
ALAT U/l	21.9 ± 4.4
TSH, mIU/L	1.8 ± 0.6
Free thyroxin, pM	17.4 ± 1.8
Vitamin D, nM	69.9 ± 21.1

BMI, body mass index; ASAT, aspartate aminotransferase; ALAT, alanine aminotransferase; TSH, thyroid-stimulating hormone.

### Effect of Fluvastatin on Lipid Profile

**Figure 2:**
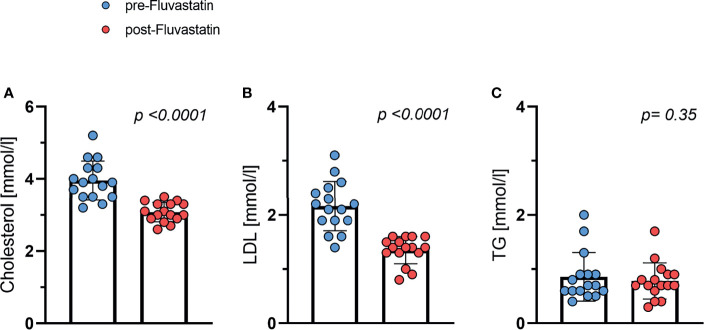
Effect of fluvastatin treatment on serum lipid levels. **(A)** Total cholesterol *p* < 0.0001 **(B)** LDL-cholesterol *p* < 0.0001 **(C)** Triglycerides (TG) *p* = 0.35.

As expected, the concentration of low-density lipoprotein (LDL) and cholesterol dropped significantly after two weeks of statin intervention. Baseline total cholesterol levels fell from 3.96 mmol/l to 3.08 mmol/l (p <0.001) (**Figure 2A**). The concentration of LDL cholesterol decreased from 2.16 mmol/l to 1.35 mmol/l (p <0.001) (**Figure 2B**). The triglyceride concentration remained stable (**Figure 2C**) indicating that the study medication was taken as prescribed. The study medication was well tolerated by all study participants and no adverse events related to the study medication were observed.

### Oral Glucose Tolerance Test

**Figure 3:**
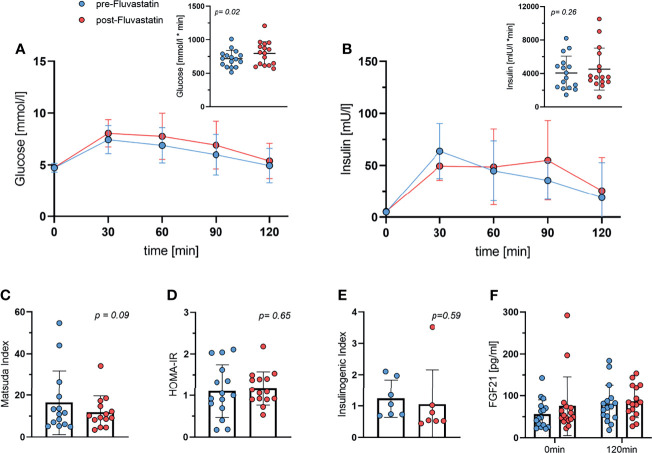
Oral Glucose tolerance test. **(A)** Glucose during oGTT both before and after two weeks of treatment with fluvastatin and respective area under the curve, *p* = 0.02. **(B)** Insulin levels during oGTT and area under the curve, *p* = 0.26. **(C)** Matsuda Index, *p* = 0.09. **(D)** HOMA-IR, *p* = 0.65. **(E)** Insulinogenic Index; *p* = 0.59. **(F)** FGF21 concentration at baseline (0min) and at the end (2h) of the oGTT both before and after two weeks of fluvastatin administration. Effect of time after ingestion of glucose *p* = 0.01, effect of fluvastatin *p* = 0.2, interaction between time x fluvastatin *p* = 0.47.

In order to assess the effect of fluvastatin on glucose tolerance and insulin sensitivity, we performed an oGTT, both before and after two weeks of fluvastatin intake. The administration of fluvastatin had a significant effect on the blood glucose concentration during oGTT, as determined by area-under-the-curve (AUC). The AUC during oGTT increased from baseline 717 ± 125 to 796 ± 177 mmol/l x min (p=0.02). Mixed-effects analysis of the glucose concentrations showed a significant effect of time after ingestion of glucose (p<0.0001) and for fluvastatin (p=0.045). There was no significant interaction between time and fluvastatin (p=0.23) (**Figure 3A**). To corroborate our analysis, we also performed a two-way ANOVA, excluding subjects with missing values and obtaining virtually the same result. Effect of time on glucose concentration (p<0.0001) and administration of fluvastatin (p=0.036) were still significant, while the interaction between time and fluvastatin remained insignificant (p=0.13). The AUC for insulin was 4069 ± 1992 mU/l x min at baseline and increased to 4518 ± 2511 mU/l x min after two weeks of treatment, which was not statistically significant (p=0.26). In the mixed-effects model analysis, insulin concentration during oGTT showed a significant interaction between glucose administration and fluvastatin (p=0.04). The time after ingestion of glucose had a significant effect on insulin concentration (p<0.0001), whereas fluvastatin did not (p=0.22) (**Figure 3B**).

Fasting glucose levels remained unchanged at 4.7 mmol/l before and 4.8 mmol/l (p=0.73) after fluvastatin treatment. Fasting insulin levels also increased only insignificantly from 5.1 mmol/l at baseline to 5.5 mmol/l (p=0.51) after two weeks of fluvastatin intake.

Next, we calculated the Matsuda index, a parameter of insulin sensitivity derived from the oGTT data (30). The Matsuda index was lower after the intake of fluvastatin (p=0.09) (**Figure 3C**), but this was not statistically significant. HOMA-IR, a surrogate parameter of insulin-resistance and β-cell function, did not change significantly (p=0.65) (**Figure 3D**). The insulinogenic index (IGI), a frequently used index of β-cell function (31), did not change significantly (p=0.59) (**Figure 3E**).

We also measured levels of FGF21, which has been shown to be associated with insulin resistance (32) and BAT activity (33). FGF21 increased significantly after administration of 75g of glucose (p=0.01 for time), but fluvastatin intake did not significantly affect FGF21 levels (p=0.2) (**Figure 3F**).

### Energy Expenditure and Respiratory Exchange Ratio

**Figure 4:**
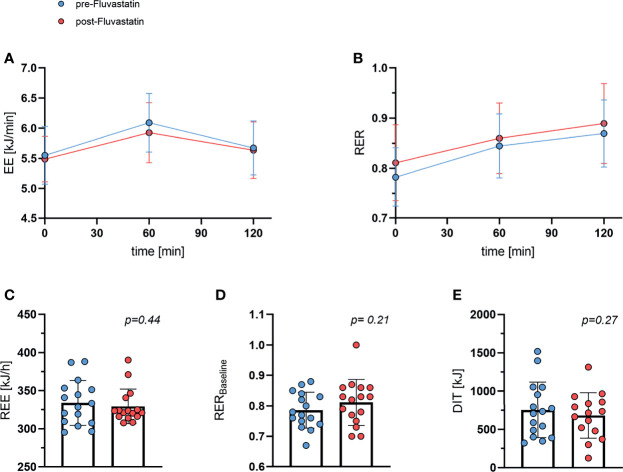
Whole body energy expenditure and respiratory exchange ratio. **(A)** Energy expenditure (EE) during oGTT. Effect of time after ingestion of glucose p < 0.0001, effect of fluvastatin *p* = 0.57, interaction between time x fluvastatin *p* = 0.21. **(B)** Respiratory exchange ratio (RER) during oGTT. Effect of time after ingestion of glucose p < 0.0001, effect of fluvastatin *p* = 0.28, interaction between time x fluvastatin *p* = 0.56. **(C)** Resting energy expenditure (REE) before and after two weeks of fluvastatin *p* = 0.44. **(D)** Baseline (0 min of oGTT) respiratory exchange ratio before and after fluvastatin *p* = 0.21. **(E)** Diet induced thermogenesis (DIT), i.e. difference between energy expenditure at baseline *vs.* 60 min after consuming 75g of glucose *p* = 0.27.

As DIT contributes to the overall energy balance, we investigated whether fluvastatin affected the increase in EE after intake of glucose. EE increased from 5.55 ± 0.48 kJ/min to 6.09 ± 0.49 kJ/min (one hour) and to 5.68 ± 0.45 kJ/min (two hours post glucose uptake) before exposure to fluvastatin. After two weeks of treatment with fluvastatin EE at baseline was 5.49 ± 0.38 kJ/min and increased to 5.93 ± 0.50 kJ/min (1h) and 5.64 ± 0.47 kJ/min (2h). As expected, the effect of glucose was highly significant (p<0.0001) while fluvastatin had no significant influence on EE (p=0.57); there was no significant interaction between glucose administration and fluvastatin (p=0.21) (**Figure 4A**). In addition, the respiratory exchange ratio (RER) increased from 0.78 ± 0.06 to 0.84 ± 0.06 (1h) and 0.87 ± 0.07 (2h) before administration of fluvastatin. After treatment, RER was 0.81 ± 0.08 at baseline and increased to 0.86 ± 0.07 (1h) and 0.89 ± 0.08 (2h). As with EE, the effect of glucose administration was significant (p<0.0001) while fluvastatin did not affect RER (p=0.28) and did not interact with glucose administration (p=0.56) (**Figure 4B**). Multiple comparison revealed that the effect of glucose from baseline to 1h and baseline to 2h was significant (p<0.0001), while the effect of glucose on RER from 1h to 2h was not significant (p=0.18).

REE remained practically unchanged from 334 kJ/h ±29 kJ/h to 329 ± 23 kJ/h (p=0.44) (**Figure 4C**). Baseline RER was lower before than after fluvastatin, 0.79 and 0.81, respectively, but the difference was not statistically significant (p=0.21) (**Figure 4D**). DIT decreased from baseline 754 ± 363 kJ to 682 ± 297 kJ after exposure to fluvastatin, but the difference was not significant (p=0.27) (**Figure 4E**).

**Figure 5:**
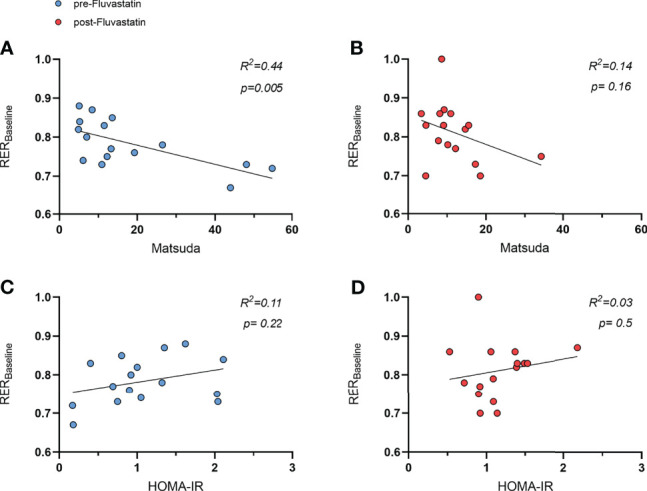
Relation of respiratory exchange ratio (RER) and indices of insulin sensitivity. **(A)** Relation of Matsuda index to RER before intake of fluvastatin, R² = 0.44, *p* = 0.005 and **(B)** after two weeks of fluvastatin, R² = 0.14, *p* = 0.16. HOMA-IR before **(C)** and after two weeks **(D)** of fluvastatin, R² = 0.11, *p* = 0.22 and R² = 0.03, *p* = 0.5, respectively.

Interestingly, at baseline the Matsuda index and RER correlated inversely (R^2 =^ 0.44, p=0.005) (**Figure 5A**). After intake of fluvastatin the tendency was still present, but the correlation was no longer significant (R^2 =^ 0.14, p=0.16) (**Figure 5B**). HOMA-IR did not show any relation to RER before (R^2 =^ 0.11, p=0.22) and after (R^2 =^ 0.03, p=0.5) fluvastatin (**Figures 5C, D**).

### BAT Activity

**Figure 6:**
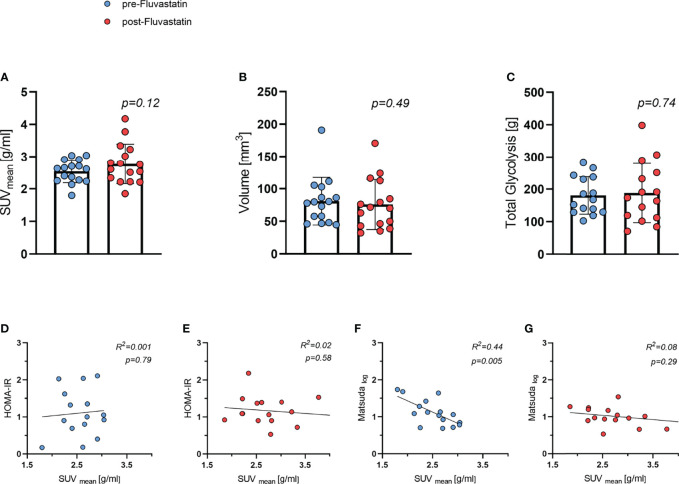
Brown adipose tissue (BAT) activity and relation to insulin sensitivity both before and after two weeks of fluvastatin treatment. **(A)** Mean standard uptake value (SUV_mean_) *p* = 0.12. **(B)** Volume of BAT *p* = 0.49. **(C)** Total lesion glycolysis *p* = 0.74. **(D)** HOMA-IR and SUV_mean_ before R^2^ = 0.001, *p* = 0.79 and **(E)** after fluvastatin intake R^2^ = 0.02, *p* = 0.58. **(F)** Matsuda and SUV_mean_ before R^2^ = 0.44, *p* = 0.005 and **(G)** after fluvastatin treatment R^2^ = 0.08, *p* = 0.29.

As part of the main study protocol and based on the established BARCIST (Brown Adipose Reporting Criteria in Imaging Studies) criteria (34), we evaluated BAT activity after stimulation with cold and the β3-agonist mirabegron. The mean standardized uptake value (SUV_mean_), volume of BAT, and total lesion glycolysis were used to evaluate BAT function in FDG-PET. Both SUV_mean_ of [^18^F]-FDG into BAT and volume of BAT did not differ significantly before and after treatment, 2.54 g/ml *vs.* 2.77 g/ml (p=0.12) (**Figure 6A**) and 80.9 cm³ *vs.* 75.8cm^3^ (p=0.49) (**Figure 6B**), respectively. Also, BAT total lesion glycolysis was similar 182 *vs.* 189 g (p=0.74) (**Figure 6C**). We next investigated whether parameters of insulin sensitivity were associated with BAT activity. There was no correlation between HOMA-IR and SUV_mean_ (**Figures 6D, E**). At baseline, BAT activity (SUV_mean_) was inversely related to the Matsuda Index (R^2^ = 0.44, p=0.005) (**Figure 6F**). After two weeks of treatment with fluvastatin however, this inverse correlation (SUV_mean_/Matsuda Index) was no longer present (R^2^ = 0.08, p=0.29) (**Figure 6G**).

### Core Body Temperature

As BAT is an important contributor to temperature homeostasis, we also measured the core temperature before and after cooling at each visit. During cooling, temperature dropped from 36.56 ± 0.29°C to 36.34 ± 0.21°C before fluvastatin administration and decreased also from 36.48 ± 0.27°C to 36.28 ± 0.2°C after two weeks of fluvastatin. Cooling had a significant effect on core temperature (p = 0.005), whereas the effect of fluvastatin was not significant (p=0.052). Fluvastatin and cold exposure did not interact (p=0.65).

## Discussion

Treatment with statins has greatly reduced cardiovascular morbidity and mortality, both in secondary (35) and primary prevention (8) by reducing total cholesterol and LDL-cholesterol levels. Data from both observational studies and clinical trials indicate that statin therapy is associated with an increased risk of diabetes mellitus (7, 8). However, the molecular mechanisms behind this association are not yet fully understood. In this study, we examined the effect of two weeks of full dose fluvastatin on glucose tolerance, insulin sensitivity and BAT activity in healthy young male volunteers.

Two weeks of fluvastatin treatment increased the AUC of glucose during an oGTT, which was paralleled by trend to higher insulin AUC. No evidence was found that the decrease in insulin sensitivity was due to impaired cold-induced BAT function.

We had previously described reduced browning of white adipose tissue in mice and reduced probability of active BAT on routine FDG-PET/CT scans in patients taking statins as opposed to patients without statin treatment (10). BAT in adult humans had been rediscovered (36) due to its rapid uptake of FDG in routine FDG-PET/CT scans, suggesting that glucose disposal is an important function of BAT. Indeed, recent data demonstrates that glucose (37) and triglyceride-rich lipoproteins (38) are the main substrates for BAT in mice (37). Also, disruption of glucose transporter type 4 (GLUT-4) translocation in murine brown adipocytes results in insulin resistance (39). In cross-sectional studies, based on routine FDG-PET/CT scans, presence of active BAT was generally associated with a metabolically favorable phenotype and a lower incidence of diabetes (19, 40). Consequently, we studied whether the higher levels of glucose were associated with reduced BAT activity: Overall, treatment with fluvastatin did not lead to a significant change in cold-induced BAT activity as determined by FDG-PET. In addition, we did not detect any association between cold-induced BAT metabolic activity and glucose tolerance on an individual level. However, this does not rule out the possibility that molecular changes influence substrate uptake by brown adipocytes in the non-cold stimulated state. We had observed that several markers of human BAT, such as UCP1, CIDEA, CPT1B and ELOVL3, were expressed at lower levels after fluvastatin treatment, which corroborated findings from animal models (10). It should be noted that glucose uptake into BAT can also be stimulated by insulin (41), underscoring its possible role as a “glucose sink” in the post-absorptive state. Intriguingly, in our cohort levels of GLUT-4 but not GLUT-1 in supraclavicular BAT were reduced on the mRNA level after fluvastatin treatment (10).

It might seem counterintuitive that the two parameters Matsuda index and SUV_mean_ displayed an inverse correlation at baseline. This indicated that subjects with higher insulin sensitivity actually showed lower uptake of [^18^F]-FDG into supraclavicular BAT. However, it should be noted that our study comprised exclusively healthy, relatively young men who exercised regularly. In a similar cohort of young, healthy men with normal body weight no correlation between cold induced BAT activity and insulin sensitivity, as measured by the hyperinsulinemic-euglycemic clamp, could be detected (42). Regular endurance training as well as moderate short term exercise training have both been shown to reduce BAT activity in healthy volunteers while improving insulin sensitivity (43, 44). Thus, glucose tolerance in our participants was probably determined to a great extent by skeletal muscle. After administration of fluvastatin, we did not observe the inverse correlation between BAT activity and glucose tolerance anymore. We therefore hypothesize that statin treatment might influence insulin sensitivity of skeletal muscle rather than BAT.

In line with this notion, a previous trial showed that 10 patients with known hypercholesterolemia treated with simvastatin at different doses for at least 12 months had a significantly higher glucose AUC when compared to a matched control group. Similar to our study, fasting plasma glucose and insulin concentrations did not differ between both groups. The authors attributed the reduced glucose tolerance to decreased oxidative phosphorylation in muscle mitochondria (45) as well as to reduced lipid uptake into muscle (46).

In mice, simvastatin at clinical doses decreased glucose uptake and reduced insulin sensitivity in muscle cells through inhibition of the insulin dependent GLUT-4 expression (47). GLUT-4 in muscle is pivotal for glucose tolerance and heterozygous knockout mice develop muscle insulin resistance and diabetes (48). In mice, fluvastatin also reduced GLUT-4 expression in BAT (10). Unfortunately, we do not have data on GLUT-4 expression in human muscle in our study.

In addition to the Matsuda index, we also calculated HOMA-IR as well as the insulinogenic index and measured FGF21 levels. In contrast to the Matsuda index, HOMA-IR did not differ between baseline and fluvastatin treatment. This is not surprising as HOMA-IR only uses fasting insulin and glucose levels, which were not different between the study visits. The fact that the insulinogenic index was similar indicates no effect of statins on insulin secretion.

Other studies could not demonstrate an effect of statins on glucose tolerance. However, these studies only compared fasting blood glucose and insulin levels (49–51) or did not compare their results to a healthy control group (52).

FGF21 has been described as a potential biomarker of BAT (33). FGF21 levels did not change with fluvastatin administration in line with the comparable BAT activity before and after exposure to fluvastatin. FGF21 levels increased significantly during oGTT which is not surprising since FGF21 is regulated by insulin. High baseline FGF21 levels are associated insulin resistance (53), which we did not observe in our cohort.

Taken together, our findings indicate that treatment with statins reduces insulin sensitivity rather than insulin secretion.

In addition to parameters of glucose sensitivity, we determined DIT by indirect calorimetry. DIT has been described as a major function of BAT (54–56), although this has been contested (57, 58). DIT was not influenced by fluvastatin.

We observed a negative relation between the Matsuda index and the baseline RER before treatment with fluvastatin. RER is calculated as a dimensionless ratio of endogenous carbon dioxide production and oxygen consumption measured by indirect calorimetry. The value of the RER indicates which macronutrients are metabolized (59). Interestingly, the correlation could no longer be observed after fluvastatin administration.

Our study is limited by the following points: As our trial was primarily designed to assess BAT function after statin exposure, glucose tolerance was a secondary endpoint. Due to mild effects of statins on glucose tolerance in healthy individuals, the cohort size was not sufficient to detect potential small effects on insulin secretion. Our study is the first to compare the effect of statins on glucose tolerance with BAT activity in healthy human volunteers. However, it should be noted that BAT glucose uptake after simulation by cold and β_3_-adrenoreceptor agonists differs from stimulation of the tissue by insulin (41). At the time when the trial was planned activation of BAT by the β_3_-adrenoreceptor agonist mirabegron had been described as a promising way to activate human BAT. However, more recent data indicates that human BAT function is driven by the β_2_-adrenoreceptor (60).

Moreover, our study cohort was very insulin sensitive and does not reflect the typical patient population taking statins for primary or secondary cardiovascular prevention. However, the degree of change in glycaemia in patients taking statins is usually also very mild with an increase of 0.1 percentage points in glycated hemoglobin levels in clinical trials (8). Strengths of our study comprise the fact that we pre-screened participants for cold-induced thermogenesis before including them into the trial. We used state-of-the-art measurements of BAT activity with FDG/PET-MRI and calorimetry. Our standardized protocol limited potential confounders otherwise introduced by inter-device or inter-protocol variability.

In conclusion, two weeks of fluvastatin treatment slightly reduced glucose tolerance in healthy male volunteers. This finding was not due to changes in cold-induced BAT activity. However, the insulin stimulated glucose uptake into BAT at thermoneutral temperatures might play a role. Further, it might be caused by changes in muscle and thus warrants further studies.

## Data Availability Statement

The raw data supporting the conclusions of this article will be made available by the authors, without undue reservation.

## Ethics Statement

The studies involving human participants were reviewed and approved by Ethikkommission Nordwest- und Zentralschweiz, University of Basel. The patients/participants provided their written informed consent to participate in this study.

## Author Contributions

MJB: Conceived the study, performed experiments, analyzed data, drafted the manuscript. MF: analyzed data, drafted the manuscript. CM, JS, GG, JM, and AB: performed experiments. MB: performed experiments, analyzed data. IB and CW: conceived the study, analyzed data. All authors contributed to the article, critically revised and approved the submitted version.

## Funding

The study was supported by the Swiss National Science Foundation (Grant No. PZ00P3_167823) and grants from the Bangerter Rhyner Foundation Basel, the Nora van der Meeuwen Foundation Basel, and the Medical Faculty of the University of Basel to MJB. The sponsors had no role in the design, conduct, or analysis of the study.

## Conflict of Interest

The authors declare that the research was conducted in the absence of any commercial or financial relationships that could be construed as a potential conflict of interest.

## Publisher’s Note

All claims expressed in this article are solely those of the authors and do not necessarily represent those of their affiliated organizations, or those of the publisher, the editors and the reviewers. Any product that may be evaluated in this article, or claim that may be made by its manufacturer, is not guaranteed or endorsed by the publisher.

## Abbreviations

AT, Adipose tissue; BAT, Brown adipose tissue; AUC, Area under the curve; CIT, Cold induced thermogenesis; DIT, Diet induced thermogenesis; EE, Energy expenditure; RER, Respiratory exchange ratio; SUV_mean_, Mean standard uptake value; OGTT, Oral glucose tolerance test; REE, Resting energy expenditure.
